# Design Decisions for Wearable EEG to Detect Motor Imagery Movements

**DOI:** 10.3390/s24154763

**Published:** 2024-07-23

**Authors:** Ana Carretero, Alvaro Araujo

**Affiliations:** B105 Electronic Systems Lab, ETSI de Telecomunicación, Universidad Politécnica de Madrid, 28040 Madrid, Spain; araujo@b105.upm.es

**Keywords:** design decisions, detection, EEG, imagery motion patterns, wearable

## Abstract

The objective of this study was to make informed decisions regarding the design of wearable electroencephalography (wearable EEG) for the detection of motor imagery movements based on testing the critical features for the development of wearable EEG. Three datasets were utilized to determine the optimal acquisition frequency. The brain zones implicated in motor imagery movement were analyzed, with the aim of improving wearable-EEG comfort and portability. Two detection algorithms with different configurations were implemented. The detection output was classified using a tool with various classifiers. The results were categorized into three groups to discern differences between general hand movements and no movement; specific movements and no movement; and specific movements and other specific movements (between five different finger movements and no movement). Testing was conducted on the sampling frequencies, trials, number of electrodes, algorithms, and their parameters. The preferred algorithm was determined to be the FastICACorr algorithm with 20 components. The optimal sampling frequency is 1 kHz to avoid adding excessive noise and to ensure efficient handling. Twenty trials are deemed sufficient for training, and the number of electrodes will range from one to three, depending on the wearable EEG’s ability to handle the algorithm parameters with good performance.

## 1. Introduction

Electroencephalography (EEG) is a method that records brain signals, and it is used in many configurations and applications, such as epilepsy, sleep disorders, brain–computer interfaces (BCIs), and mental states.

The way to obtain these signals is through an electrode cap that covers the head and allows the attachment of the electrodes to the scalp. There are currently many electrode caps that are available on the market [1], the main limitations of which are their size and weight, as these characteristics make them uncomfortable for users and, in some cases, unportable.

These devices do not allow for complete monitoring throughout the day; thus, clinical treatments are restricted, and the integration with actuators in a closed-loop system is not feasible, which is why they are regularly used in hospitals and research labs for specific tasks with limited amounts of time.

In order to avoid these limitations, wearable EEGs (wEEGs) have emerged as a solution to lead to new treatments, diagnostics, and applications, and they are expected to improve the patient’s quality of life and comfort with their treatment, as well as to make it possible to register real behaviors over the course of a day. If a small device only covers the brain zones that a doctor needs to monitor, then it could be worn for 24 h, seven days a week. In the future, it is expected that this small device will become like a wearable band-aid for recording signals that are useful for the diagnosis and treatment of certain diseases. Moreover, wearable band-aids can be used not only in the hospital but also at home, during work, or even during exercise.

In recent years, in addition to electrode caps, different wearable EEGs have appeared on the market [1,2,3,4] for various applications. The BitBrain Diadem has been used to test the user experience [5]. The B-Alert X-10 has been used to test the motivation of users when they perform specific exercises [6]. The Emotiv Insight has been used to control some external smart home devices [7] and the cursor of an application [8].

There are others, such as the emotive EPOC X, Muse S Headband, and Neurosity Crown, that have been tested for emotion recognition [9] and that use emotions (in the case of emotive EPOC X) to improve the virtual reality experience [10]. Ganglion and Cyton + Daisy are the devices that are the most similar to the conception of wearable EEG. They are used to study, among other things, motor imagery movements [11,12]. The issue with these devices is that they function as EEG interfaces, meaning that they do not process data to make decisions about the movement. It may be possible for the Ganglion to make some decisions because it has an integrated microcontroller, but not complex decisions. Data portability is limited to having a connection with a Bluetooth dongle.

Other wearable-EEG initiatives using minimal electrodes are focused on stress monitoring while driving to obtain real-execution-time responses without the need for calibration [13,14]; additionally, emotional recognition has demonstrated good performance [15]. These studies offer valuable contexts for applied processing techniques.

In line with these EEG interfaces, the most recently developed wearable EEG is the Galea, which can measure the physiological response of the user when they are experiencing virtual content to obtain data from the parasympathetic and sympathetic nervous systems using more electrodes on the face around the eyes [16].

To summarize all of the technology presented, there are two recognized sides: the open-source option, led by the OpenBCI company, and the private sphere, led by BitBrain. Both reduce the number of device channels and select the ones related to the final configuration desired by the user.

The four key aspects that wearable EEG must comply with are comfort, autonomy, portability, and wireless operation, which are determined, first, according to the wearable-EEG size, with the number of electrodes being important for the wearer. Considering that this device is positioned on the head, if it is large, heavy, or has wires, it is useless because of the user’s discomfort, which is why, in this work, we did not consider caps, as they are uncomfortable and cover the head the whole time. The user could feel awkward wearing the device outside the hospital. Moreover, they are tedious and difficult to mount on and unmount from the head, not only because of their size but also because of the gels needed to obtain good contact with the scalp. The amount of gel used is directly related to the number of electrodes; thus, reducing the number of electrodes reduces the amount of gel in contact with the head and, obviously, the size of the wearable EEG. Consequently, working with wearable EEGs as small as can be created will reduce these inconveniences.

In addition, there are computational and memory limitations because of power consumption, which means that the sampling frequency and processing algorithm need to be carefully selected. These last two aspects involve a trade-off between the size of the wearable EEG device and its computational capacity. The balance in this trade-off is possible because many electrodes are not needed to obtain a good performance; however, the computational load has to be strong enough to support complex algorithms [17].

In this work, we focused on the BCIs for motor imagery movements because this type of signal can benefit not only individuals who are mobile but also those who are not. This is useful for people who do not have a continuous neural connection between the brain and one or more limbs (due to spinal cord injury). After detection, the device knows the movement that the person wants to execute; then, this information can be delivered to the specific muscles and nerves required to initiate the intended movement as it is thought in the brain.

Consequently, the objective of this work is to provide clarity regarding the decisions about the number of electrodes required for the wearable EEG, considering the developed algorithms and their complexities to address the computation problems. The ultimate goal is to design an optimal motor imagery acquisition device that strikes a balance between the obtained information and usability.

Because this information is sensitive, in the future, ethics and data protection will be carefully considered to ensure the security of users [18].

## 2. Materials and Methods

### 2.1. Datasets and Sampling Frequencies

We looked for datasets according to the following criteria: more than one record per subject; more than one motor imagery movement; and data recorded with different sampling frequencies.

Each dataset works with its own frequency. For this reason, in this work, three datasets were selected. We tested different user-record sampling frequencies because this limited the signal bandwidth and the number of samples to be processed, with higher frequencies allowing for more samples per subject record. Hence, more information could be extracted, and the results are more reliable. However, conversely, higher frequencies require more data-processing resources, and, furthermore, having more information does not necessarily produce better results.

These datasets will be explained from low to high sampling frequencies, which were the main reason we selected them.

#### 2.1.1. PhysioNet Dataset (PDS)

The PhysioNet database [19] contains recorded images of 109 subjects opening, closing, and relaxing their left and right fists while wearing a 64-electrode cap. There are three records of the same task for each subject.

The sampling frequency is 160 Hz, which allows for the study of the influence of the use of a low frequency to record the data, which implies a bandwidth of 80 Hz. The duration is approximately two minutes per record, which means that this dataset has 160 samples per second.

#### 2.1.2. Nature Dataset (NDS)

The Nature database contains the recorded movements of the five right-hand fingers of 13 subjects with a sampling frequency of 1 kHz, which results in more data than in the previous database, and 22 input channels (electrodes) [20].

In this case, the bandwidth is not 500 Hz but 100 Hz because a 0.53–100 Hz bandpass filter was applied to these recordings. The data between the frequencies of the filter are still sampled at 1 kHz, which implies more data than in the previous database. Specifically, there are 1000 samples per second.

The record duration is 55 min, and each trial has an average duration of three seconds: 1 s of movement signal and 1.5–2.5 s of relaxation. Although there is only one record, it can be split up because of its long duration.

#### 2.1.3. Our Own Dataset (OODS)

To test higher frequencies, our own dataset was recorded using an ad hoc PCB that is able to connect two electrodes (one in a selectable position and the reference behind the ear) [21]. The recording time is two seconds, and the sampling frequency is 9.524 kHz; thus, there are 9524 samples per second.

There are four movements that were recorded four times: the opening/closing of the left/right fists. Relaxation was also recorded ten times.

#### 2.1.4. Common Decisions in Dataset-Preprocessing Stage

Based on the assumption that every subject is different and must be treated independently [12], for the PDS and NDS, three subjects were considered, while for OODS, just one subject was considered. Moreover, 50/60 Hz artifacts were removed, and the movement signals were separated from relaxation for flexible combinations in further analyses.

### 2.2. Motor Imagery Brain Zone

The brain zones, according to the electrode positions, were selected using Brodmann areas [22]. These areas are a way of mapping the cortex and its distinguished functions. Because we worked under the motor imagery paradigm in this study, the brain zones implicated in the imagery movements were as follows: the primary motor cortex, which is the main source of motor activation; the supplementary motor area (SMA), which is involved in motor learning and planning, as well as the motor activation of the hand; and the pre-motor cortex, which also mainly relates to motor action planning. There are other sections of the brain that are involved with motor imagery movement, but their main functions are not related to it: the dorsal anterior cingulate area, which is also involved in motor action planning, and the opercular area, which is involved in the motor action of the fingers and toes. In addition, there are the angular and occipitotemporal areas, which are involved in visual processing and object recognition, which are important because most of our tests need object recognition to subsequently execute the movement.

The work in [23] was used to link the Brodmann areas related to motor imagery movement with the electrodes involved in the 10–5 electrode configuration.

For the main zones implicated in motor imagery (MIZ), the implicated electrodes are the FC3, FC2, FC4, C5, C3, C4, and C6 electrodes. For the secondary sections, the more relevant electrodes are the F7, F8, P7, and P8 electrodes. The dorsal anterior cingulate area is a depth zone and does not match with any electrode. Handedness is important in the main zone because the use of the left side of the body activates the right side of the brain and vice versa. In contrast, the use of the right side of the body activates the left side of the brain.

### 2.3. Preprocessing

The dataset signals were ready for use, and no additional filtering or preprocessing was needed.

### 2.4. Detection Algorithms

The idea is to run the algorithms inside the device within the execution time, which produces the thought-of imagery movement result in a moment (or as fast as possible). This means that there is no server to save the data, process them, and return them with the result. Consequently, the algorithm selection has to be conducted cautiously in terms of its performance.

In terms of wearable EEGs, there are some limitations in the chips that must be considered. These chips, which control the logic and process of the detection, have limited functions, a limited size, limited complexity, and a limited economic cost to satisfy wearable-EEG demands.

The selection of the algorithms was based on these premises, which assume that they have to be as simple and fast as possible, although, in fact, nowadays, many chips perform well in terms of these requirements.

Two detection algorithms were selected for implementation: the Fast Independent Component Analysis (fastICA) algorithm and the Common Spatial Pattern (CSP) algorithm. Both of them have been used in similar experiments [24].

Other techniques were used in combination with these algorithms. For the fastICA algorithm, a correlation was applied for the resultant components. For the CSP algorithm, a previous filter bank (FB) was used.

All of the developed code was implemented in MATLAB 2023.

#### 2.4.1. FastICACorr Algorithm

**Figure 1:**
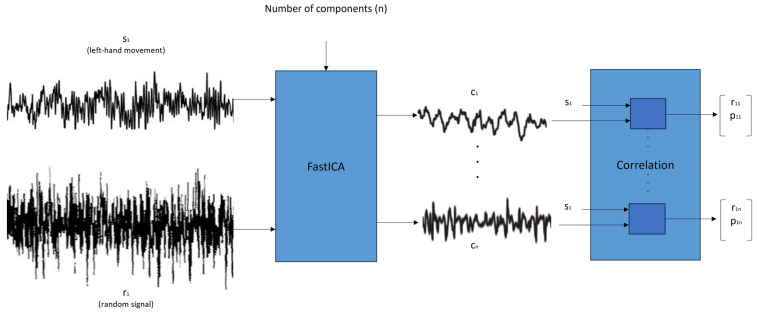
FastICACorr algorithm block representation.

Figure 1 presents the FastICACorr algorithm process, which we use to explain the two parts of the FastICACorr algorithm.

##### FastICA Algorithm

The reason we selected the FastICA algorithm is that its use can be extended to obtain characteristics from a signal. The FastICA algorithm can provide information about the differences between different signals for a subsequent comparison. Moreover, its output can be generated in a short lapse of time, and this time depends on the complexity selected when it is implemented.

In this work, we used this algorithm to separate the brain signals into all of their possible components because EEG signals are formed by many mixed signals.

The Independent Component Analysis (ICA) algorithm is based on the separation of the original source signals that conform to the treated signal. In terms of EEGs, the observed signals are the potentials presumably generated by mixing some of the underlying brain activity components.

The “fast” property is focused on finding the maximum of the non-Gaussianity, which, for this case, is measured via negentropy approximation.

All of the formal expressions for implementing the FastICA algorithm were based on those in [25]. In addition, to obtain good negentropy approximation values, the three non-quadratic functions of the reference work were implemented.

In this work, we wanted to study the performance of just one electrode as well. The input of this algorithm is a vector formed by one-person single-imagery-movement trials (s_1_ of the left-hand movement in Figure 1) with all of the electrodes to be studied in every case. For example, for 2000 samples per trial, if there are 4 trials for one person per image of open-hand movement for one electrode, then the vector will be 4 (trials) × 2000 (samples). If there are two electrodes, then the vector will be 8 × 2000. This vector was compared with a random signal (r_1_ in Figure 1) to avoid the influence of any other predefined signals.

In this way, the information of each electrode is added to the general information; thus, if the electrode addition decreases the performance, then the added electrodes are not contributing to improving the results.

The outputs of this algorithm are the “components” (from c_1_ to c_n_ in Figure 1, with n as the number of components), which can be considered unique keys decoded for every input signal that is introduced.

##### Correlation

Correlation is a technique that shows a mutual connection between two variables, and, to measure it, we used the corrcoef command in MATLAB, which outputs two matrices: the “r” matrix, which shows the correlation coefficients, and the “p” matrix, which can be used to assess the hypothesis on the relation between the observed phenomena, as seen in Figure 1.

In this work, we compared the fICA input signal with every output component obtained from the fICA. For example, if we had 20 output components (n = 20 in Figure 1), then we calculated the correlation between the fICA input signal that generated these components (s_1_ of Figure 1) and the 20 output components obtained (from c_1_ to c_20_). Thus, we had 20 pairs of r–p matrices.

These matrices are symmetrical because the order for calculating the correlation was not significant in these cases. The values on the diagonal are one because a variable always has a perfect correlation with itself. In conclusion, one paired r–p value was used per calculated correlation.

As the output and input are signals with many samples, this technique allows for a reduction from a number of samples to a pair of them.

#### 2.4.2. FB-CSSP Algorithm

**Figure 2:**
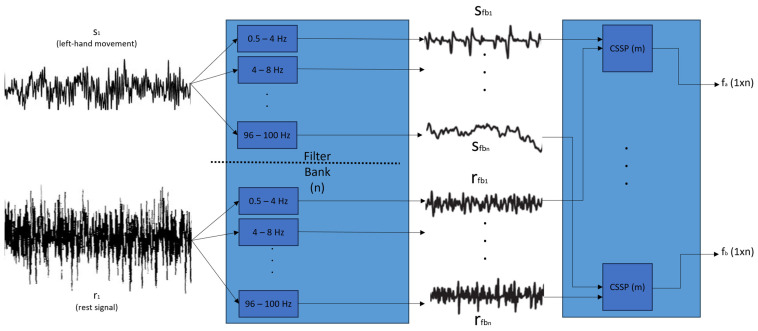
FB-CSSP algorithm block representation.

Figure 2 shows the scheme of this algorithm, which is the image that we will use for its explanation.

##### Filter Bank (FB)

A filter bank is a group of parallel filters with different frequencies and a narrow bandwidth that allows for the separation of an input signal into many different signals related to the input. The separation of some different signals is needed because, in this way, the CSSPs will have more data to build a reliable output. In addition, because the filters cover the whole frequency spectrum of each database, the algorithm generates the output using all possible frequencies, from the lowest to the highest. In the case of Figure 2, as an example to understand the process of this filter bank, the frequency limit is 100 Hz.

To obtain more samples to include in the CSP algorithm, a filter bank is proposed. This filter is formed by a group of bandpass filters that start at 0.5 Hz and increase until the database frequency limit is reached.

When selecting the bandpass filter bandwidth, we separated the frequencies into two groups: known bands and unknown bands. The known bands are the delta (0.5–4 Hz), theta (4–8 Hz), alpha (8–12 Hz), beta (12–30 Hz), and gamma (30–45 Hz) bands. The unknown bands start at 55 Hz or 65 Hz (depending on the precedence of the data) and finish at the frequency limit.

Following the assumptions of the first uses of this algorithm [26], for the known bands, sub-bands are generated with a width of 4 Hz. The sub-beta bands are as follows: 12–16 Hz; 16–20 Hz; 20–24 Hz; 24–28 Hz; 28–32 Hz. The sub-gamma bands are as follows: 30–34 Hz; 34–38 Hz; 38–42 Hz; 42–46 Hz. The delta, theta, and alpha bands are their own bands.

Among the unknown bands, as these frequencies are not classified, the width of the filters is 5 Hz for compatibility with the frequency limit.

##### Common Spatial Signal Patterns (CSSPs)

The Common Spatial Pattern algorithm was the basis for the CSSP algorithm of this study because it focuses on decreasing the dimension of the data using linear transformations. In this manner, a low-subspace projection matrix is created, the rows of which conform to the weights for the included channels. Additionally, this data reduction is controllable via the “m” parameter, which is the reduced-dimension parameter. The limitation of this algorithm is that only two different types of input signals can be tested at once.

In our work, execution with just one electrode was required, which is why this algorithm needed to be adapted. Following the basic CSP formulas in [27], in this “Signal-Patterns” algorithm, the inputs are the output signals of the filter bank that belong to some of the trials of one person performing one movement for every electrode included in the test. That is, the vector has as many input signals as the number of filters generated from the filter bank (from S_fb1_ to S_fbn_ in Figure 2). As this algorithm also needs to compare two signals, for testing, all of the electrode configurations were selected instead of only one electrode. The rationale for mixing the post-filtered electrode signals in the same execution is the same as for the FastICA algorithm: to determine the influence of the included electrodes on the result.

In the input, the comparison is between the imagery movement signals and the rest signals.

The outputs, in this case, are two vectors with comparative features between the two input signals. One vector (f_a_ from Figure 2) comes from a comparison of the S_fb1_ and r_fb1_ inputs, and the other vector (f_b_ from Figure 2) comes from a comparison of the rfb1 and S_fb1_ inputs (inverted comparison). As the outputs are concrete vectors (f_a_ (1 × n) and f_b_ (1 × n) in Figure 2, where n is the number of filters), a post-correlation technique to reduce the data output is not needed in this case.

### 2.5. Classification Algorithms

The Classification Learner tool in MATLAB was employed to classify the results from the detection algorithms [28], and 12 classification algorithms were tested to determine the one with the best fit to the included data. A k-fold cross-validation of ten was used.

Moreover, a Support Vector Machine algorithm was implemented for a simple assessment of the results.

The outputs generated by the classifier are the success percentage for two-class classification and the confusion matrix for more-than-two-class classification.

The performances of the classification algorithms used were not deeply explored, as they were primarily utilized to verify the functionality of the detection algorithms, and, moreover, they exhibited low latency. The only precondition here was to use enough data for classification using all of the classes.

## 3. Results

The results of the various tests are presented below. Due to the numerous variables involved in the experiments (types of movements, algorithm parameters, number of electrodes, etc.), the objective was to narrow them down according to the obtained results, always seeking to optimize the information/low-resource ratio. All of the experiments were performed on three subjects. Because the results were very similar, it was not necessary to increase the number of subjects.

In the preliminary tests, the FB-CSSP algorithm showed low reliability in the specificity of the movement results because every brain zone yielded the same result when comparing movement with no movement. Consequently, the FastICACorr algorithm was used to analyze the sampling frequencies for the different databases.

The results were divided into three subsections to explore the different movement concepts: general movement (all finger movements considered as a single category) was compared with no movement/rest; specific movements, involving the separation of each finger movement, were compared with no movement; and each finger movement and no movement were compared with each other. Six categories were used: thumb, index, middle, ring, and pinkie (movement), as well as rest (no movement).

### 3.1. General Movement vs. Rest

These tests were aimed at determining whether a general movement can be detected and differentiated from no movement, involving the finger and fist movements discussed in the Datasets and Sampling Frequencies Section.

#### 3.1.1. FastICACorr Algorithm

The FastICACorr algorithm allows for the obtainment of varying numbers of components, representing the output signals explained in the algorithm definition. Tests were executed for 10 and 20 components to observe the differences in the algorithm’s performance. The results obtained from one electrode (C4) and one subject for the three databases are shown in Table 1, which helped to determine the database for the subsequent tests.

The NDS was selected for all of the tests because the difference from the PDS is assumed to be marginal, and the sampling frequency of 1 kHz offers increased flexibility and more data to analyze. The results for both algorithm complexities are coherent: more complexity implies a better success rate.

**Figure 3:**
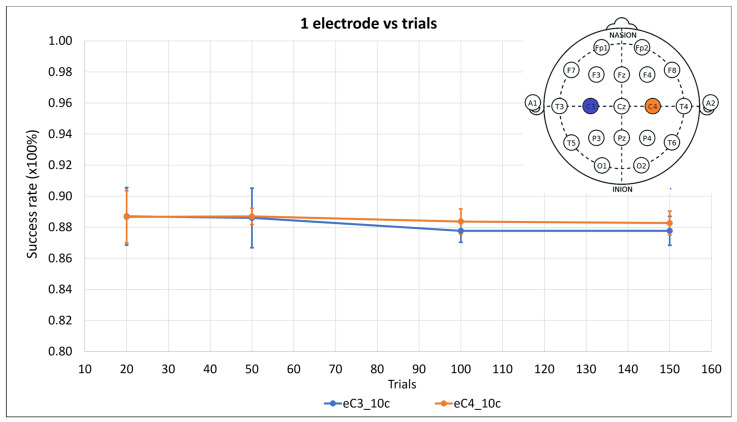
Success rate means of three subjects with their standard deviations as error bars for 20, 50, 100, and 150 trials for ten components of the FastICACorr algorithm for C3 and C4 electrodes treated independently. The positions of the electrodes on the scalp are shown.

The initial tests were for one electrode; the C3 and C4 electrodes were used because they are optimally positioned for motor imagery detection. Tests were conducted for 20, 50, 100, and 150 trials, each with ten components (Figure 3 and Table A1 of Appendix A). The aim was to assess the influence of the trials on the success rate.

In Figure 3, it is evident that the success rate decreased with 100 and 150 trials. Consequently, these trial quantities were excluded from the subsequent tests.

**Figure 4:**
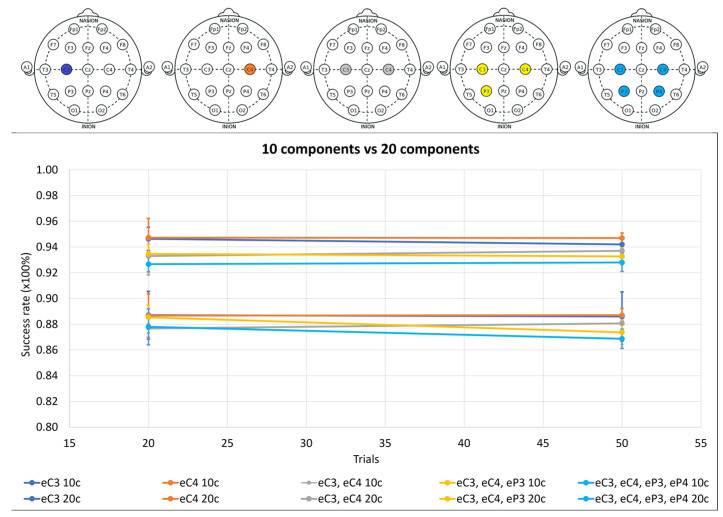
Success rate means of three subjects with error bars as standard deviations for 20 and 50 trials for one, two, three, and four electrodes. Results for ten components of the FastICACorr algorithm are denoted by “10c”. Results for 20 components of the FastICACorr algorithm are denoted by “10c”. Results for 20 components of the FastICACorr algorithm are denoted by “20c”.

Figure 4 shows the test results for one, two, three, and four electrodes for 10 components (“10c”) and 20 components (“20c”) with 20 and 50 trials. The selected electrodes were the C3, C4, P3, and P4 electrodes, maintaining proximity to the closest main zone of motor imagery detection. The aim of these tests was to assess the impact of the FastICACorr algorithm components on the success rate and the influence of increasing the number of electrodes.

When comparing the number of components in Figure 4, 20 components are considered optimal in all cases because of their higher success rates. Increasing the trial number does not consistently enhance the success rate. While there are cases in which the success rate slightly improves with 50 trials, in other cases, it worsens. Moreover, more trials imply higher performance demands.

Consequently, 20 trials are deemed the most favorable. Therefore, 20 components and 20 trials were established as constant values for the subsequent test cases. All of the relevant data are available in Table A1 and Table A2 of Appendix A.

**Figure 5:**
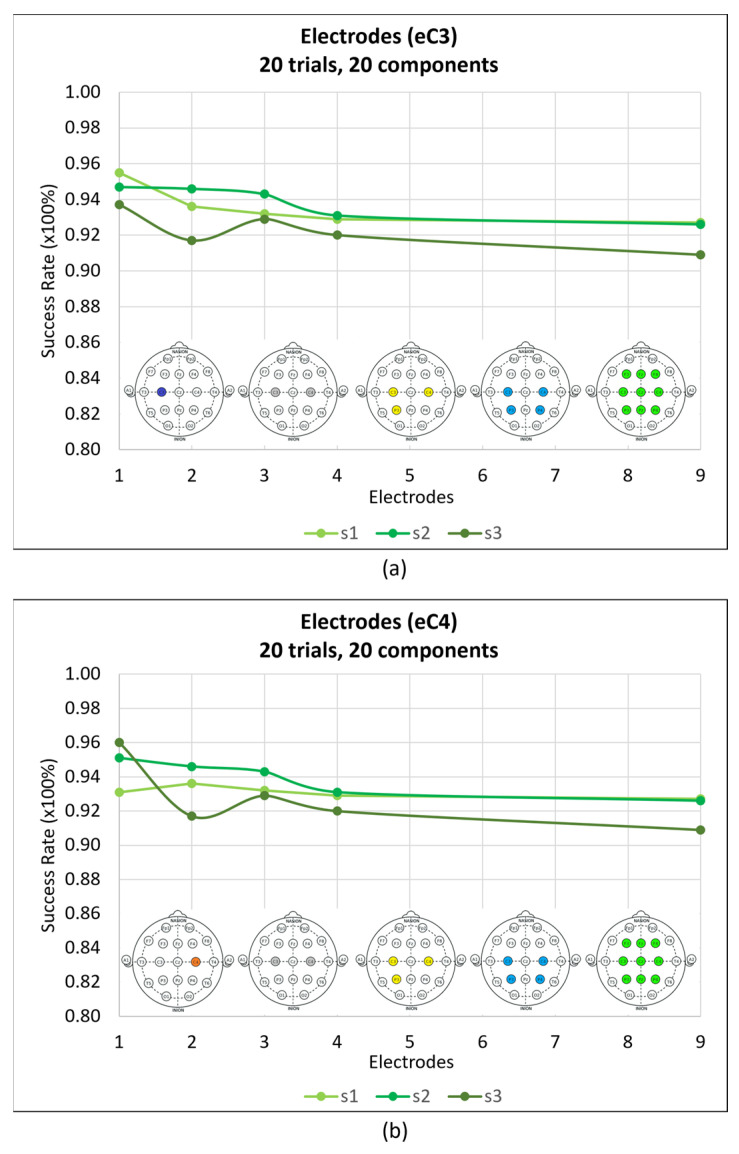
Success rates for different electrode configurations. (**a**) Electrode comparison for 20 trials, 20 components, and three subjects. Points reflect one (C3), two (C3, C4), three (C3, C4, P3), four (C3, C4, P3, P4), and nine (C3, Cz, C4, P3, Pz, P4, F3, Fz, and F4) electrodes. The headings below the subject lines contain the corresponding electrodes. (**b**) The same as (**a**); however, in this case, the first electrode is C4.

To identify the minimum number of electrodes needed to provide sufficient information for achieving a good performance, nine electrodes were tested: the C3, Cz, C4, P3, Pz, P4, F3, Fz, and F4 electrodes. The results are illustrated in Figure 5, alongside the outcomes for one, two, three, and four electrodes (Table A1, Table A2 and Table A3 of Appendix A). The first electrode was C3 (Figure 5a), as movement is imagined for the right hand, but the influence of the movement on the right zone of the brain was also assessed (Figure 5b).

##### Algorithm Complexity

**Figure 6:**
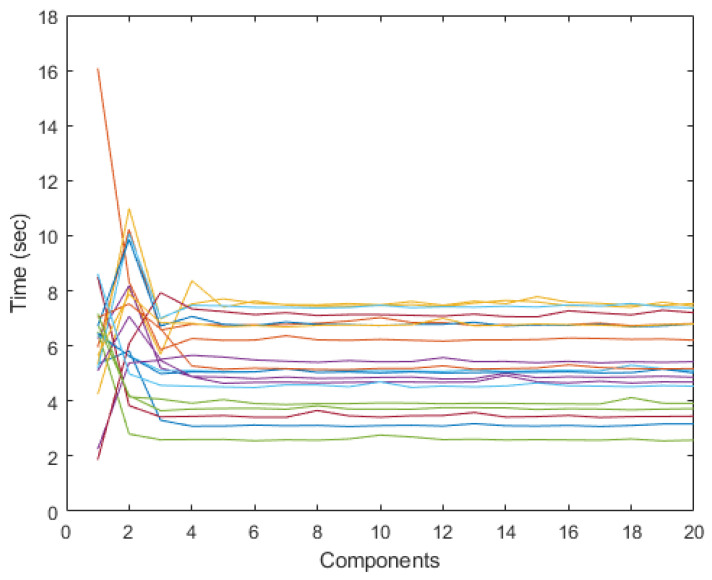
Time in seconds for each of the 20 components for 20 trials. Every color represents one component.

As this algorithm incorporates a parameter that controls the number of generated components, the time taken to obtain each component was examined. Figure 6 illustrates the time taken to obtain each component from 1 to 20, being irrelevant the relation between the color-component. The consistent results across the different trial groups, electrodes, and movement scenarios are indicative of the generic behavior of the FastICACorr components.

##### Brain Zone Tests

This subsection describes the tests aimed at determining the significance of the correct zone selection for detecting motor imagery movement. The zone has to be as small and consolidated as possible to meet the wearable-EEG requirements.

The motor imagery zones were tested with two and three electrodes, incorporating not only electrodes belonging to the MIZ but also those randomly selected from other scalp zones (the far-away zones (FAZs)). Testing the FAZs reinforces the importance of the zones and enhances the algorithm’s reliability.

**Figure 7:**
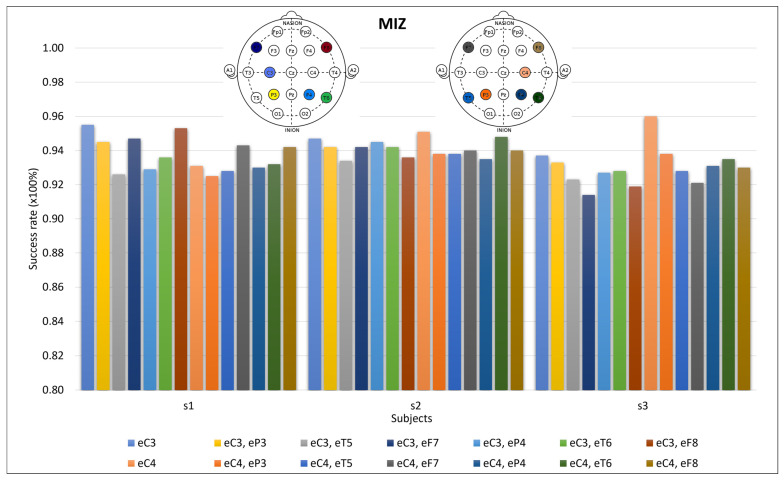
Success rates for the angular area and occipitotemporal area in conjunction with the main motor imagery zone. The first eight are related to the C3 electrode (positions on the electrode-filled head on the upper left), and the other eight are related to the C4 electrode (positions on the electrode-filled head on the upper right). The C3 and C4 electrodes are shown for comparison with the other lines.

Following the motor imagery brain zone, because the NDS electrode configuration is a 10–20-electrode system, the object recognition zones (P7 and P8) were covered by the T5 and P3 electrodes in the left hemisphere and by the T6 and P4 electrodes in the right hemisphere. The F7 and F8 electrodes were also tested for the influence of the finger motor execution zone in the imagery. In conclusion, the C3 and C4 electrodes were tested in cooperation with the P3 and P4, T5 and T6, and F7 and F8 electrodes for 20 trials and 20 components (Figure 7). The graph also presents the results for the C3 and C4 electrodes to allow for a comparison with the electrode addition.

**Figure 8:**
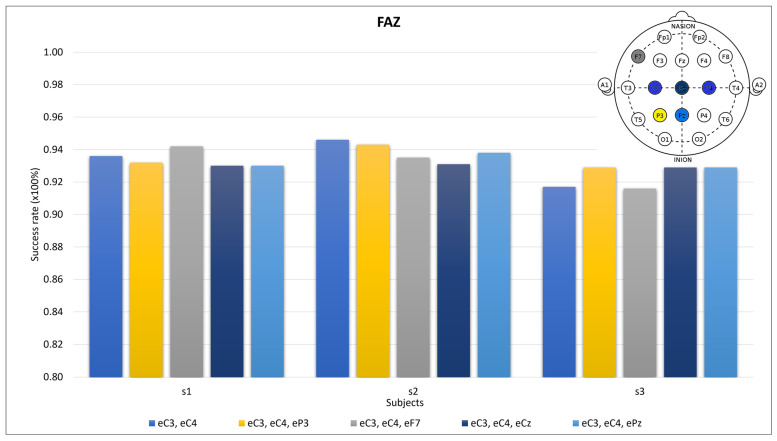
Success rates for far-away zones (FAZs) (last two blue bars) and the main motor imagery zone (first blue, yellow, and gray bars). The electrode-filled head on the upper right reflects the positions.

For the FAZs, we selected the Cz and Pz electrodes. For comparison, the overall best results across the three subjects in Figure 5 were obtained with the following groups: C3, C4, P3; C3, C4, F7; and C3, C4, T6. The C3 and C4 electrodes were selected to compare the electrode addition. All of the groups are shown in Figure 8 for 20 components and 20 trials.

Following the electrode analysis, tests for the other algorithms were executed.

#### 3.1.2. FB-CSSP Algorithm

To determine the optimal algorithm for our study, tests were executed for the FB-CSSP algorithm with one, two, and three electrodes, using 20 and 150 trials, as shown in Table 2. These parameters facilitated a comparison with the results of similar tests using the FastICACorr algorithm.

The configurable “m” parameter (reduced-dimension parameter) was set to one for all of the tests, as setting it to two and five resulted in decreased success rates.

##### Brain Zone Tests

The motor imagery zones were examined by combining the MIZ and FAZs in the tests featuring 20 trials and two and four electrodes (Table 3).

### 3.2. Specific Movement vs. No Movement

We used specific-movement-vs.-no-movement tests to differentiate the finger movements from no movement.

#### 3.2.1. FastICACorr Algorithm

The five right-handed NDS finger movements (thumb, index, middle, ring, and pinkie) were assessed and compared to no movement.

**Figure 9:**
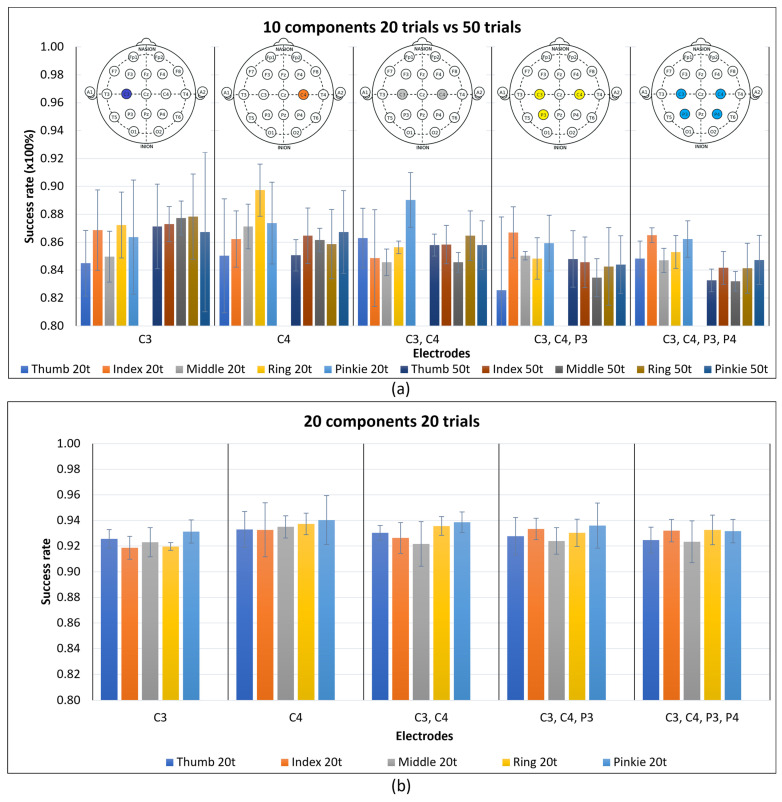
Five finger movements vs. no movement. Mean success rates of three subjects with their standard deviations as error bars are shown. (**a**) Ten components for 20 and 50 trials for the five groups of electrodes selected. (**b**) Twenty components for 20 and 50 trials for the five groups of electrodes selected.

Success rates were obtained for one, two, three, and four electrodes with 10 and 20 components and 20 and 50 trials, as reflected in Figure 9.

#### 3.2.2. FB-CSSP Algorithm

Tests were performed for the C3 and C4 electrodes individually for 20 and 150 trials, with the “m” parameter set to one. The results are presented in Table 4 and Table 5. The idea was to test the algorithm’s influence in the trials and the capability of detecting the five finger movements.

### 3.3. Specific Movement vs. Specific Movement

These tests were aimed at distinguishing a movement when all possible movements were grouped together, including no movement.

#### FastICACorr Algorithm

The finger movements were compared with each other, resulting in 15 distinct comparisons: thumb–index; thumb–middle; thumb–ring; thumb–pinkie; index–middle; index–ring; index–pinkie; middle–ring; middle–pinkie; ring–pinkie; rest–thumb; rest–index; rest–middle; rest–ring; and rest–pinkie. Confusion matrices are used to present this information, as they offer a clear and organized representation of the movement success rates, along with the percentages indicating instances when another movement was incorrectly recognized.

**Figure 10:**
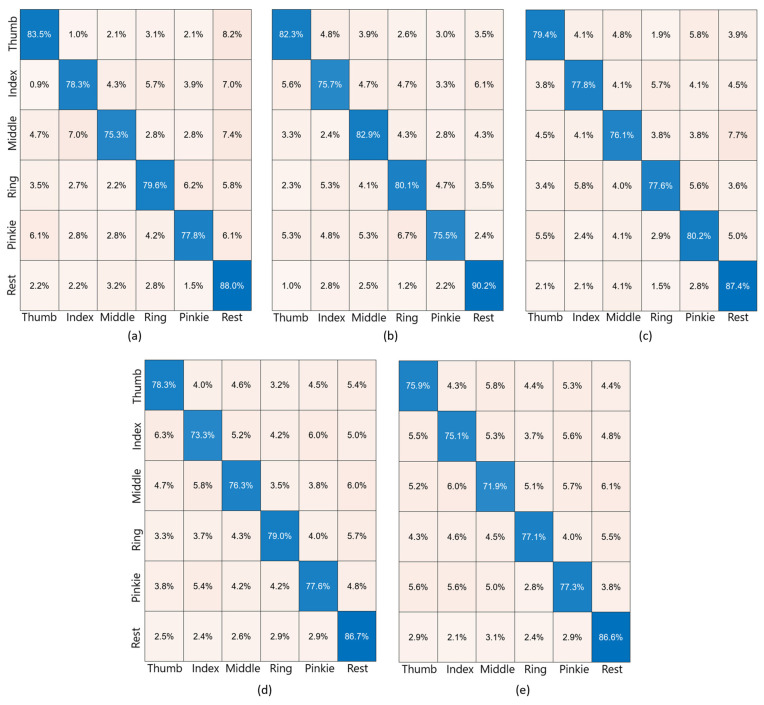
Confusion matrices for specific movement vs. specific movement for 20 trials and 20 components. The movements range from thumb movement to rest. Every box shows the success rate (in percentage) when compared one by one. Blue diagonal boxes show the proportion of times when the movement was correctly detected. Nude pink boxes show the proportion of times that the main movement was wrongly detected as the movement in the nude pink box. (**a**) C3 electrode; (**b**) C4 electrode; (**c**) C3 and C4 electrodes; (**d**) C3, C4, and P3 electrodes; (**e**) C3, C4, P3, and P4 electrodes.

The executed tests were consistent with those in the previous section. The results for 20 components and 20 trials are shown for one, two, three, and four electrodes. For simplicity, the confusion matrices for one subject are presented in Figure 10.

## 4. Discussion

The idea of developing wearable EEG comes from the need for a small device with a band-aid size that can be used for 24 h, 7 days a week. This system will be very useful to doctors to improve the diagnosis and treatment of some mental health conditions. Moreover, it will collect some data that are not currently collected because of the new cases in which these devices can be used. Furthermore, these decisions are expected to happen within a shorter lapse of time, which is the reason for studying the algorithm performance to fix the wearable-EEG specifications.

To explain the decisions for these wearable-EEG specifications, this section is divided into sub-stages for clarity and organization.

### 4.1. Sampling Frequency

The sampling frequency is an important consideration in a system because higher frequencies imply higher system performance requirements, such as computational costs and battery consumption. The minimum operating frequency value allows for improvement and the extension of the wearable-EEG life.

Observing Table 1, the PDS with 160 Hz is the dataset with the best results. The second-best dataset, very close to the PDS, is the NDS with 1 kHz, and the third-best dataset is OODS with approximately 9.5 kHz. This implies that lower frequencies improve the results due to the lower number of samples. An excess of data could lead to noise and misunderstandings for the algorithms, consequently reducing the success rate.

While one sample per 6.25 ms is sufficient for good results, having more samples provides the flexibility to work on, implement, and ensure reliable algorithm results.

The difference between the best two database success rates is less than 2%, and, in line with the points discussed above, the selected frequency was 1 kHz for the NDS.

### 4.2. Detection Algorithms

The FastICACorr algorithm was capable of extracting differentiable features from a signal in all of the cases presented, making it suitable for real-world wearable-EEG scenarios.

The success rates improve when the number of obtained components increases. However, the main drawback of this algorithm is its high computational cost in terms of the time required to obtain these components. When the computational resources are reduced, the time required to obtain the components increases.

Observing the processing times in Figure 6, obtaining a component takes two–eight seconds, stabilizing after the fourth component. These first four components are highly variable, depending on the signal. For higher numbers of components, the time is constant and quantifiable.

The numbers of trials and algorithm components were also studied, as these two parameters directly influence the computational cost of this algorithm. In Figure 3, the success rate evolution shows no improvement when the number of trials is more than 50. This can be explained by the saturation of the data and the inclusion of noise when the upper success rate limit is reached. Thus, 20 and 50 trials are preferable.

Delving into the selection of 20 or 50 trials, Figure 4 shows that although 50 trials may seem better, the increase from 20 to 50 was not observed consistently in all subjects and all electrodes, and in some cases, it was the opposite.

Regarding the number of components, according to Figure 4, using twenty components is always better than using ten, and the increase in the success rate justifies the extra performance cost that this imposes on the system.

As the performance is an important consideration for this study, and it must be balanced with the extra cost of the algorithm components, it is preferable to have fewer trials and more algorithm components, which is why, for the results in Figure 4, all of the tests were executed in advance for 20 trials and 20 components.

Longer processing times are acceptable if they are spent in the training phase of the system. After that, the device will have been trained on every movement and non-movement; thus, it will not take long to obtain the result for a new incoming signal.

The FB-CSSP algorithm is a low-computational-cost algorithm in terms of the time that it takes to finish processing. There are two reasons why this algorithm is not useful for this work. First, it is an algorithm based on a comparison of two types of signals, and the features obtained are based on this comparison. For example, movement one and movement two will have characteristics based on this comparison; however, if the detection of another movement from a random signal is wanted, comparisons with all of the different saved signals have to be performed. The second reason is that this algorithm shows the same percentage, 100%, with every electrode on the scalp because the difference between movement and no movement at the output is still high at every point. Moreover, when a specific movement is trying to be recognized, the success rate is low in comparison with that of the FastICACorr algorithm. These characteristics make the algorithm less specific for movement differentiation. Additionally, the tree structure that it generates with a random signal input with many possible movements produces an increase in the time–computational costs. Nevertheless, this algorithm is useful for other applications that fit with its characteristics.

All of these points led to the selection of the FastICACorr algorithm as the preferable algorithm. However, online tests in real time need to be conducted to corroborate its use for wearable devices.

### 4.3. Motor Imagery Brain Zone

Primary-motor-cortex and supplementary-motor-area data from (20 trials, 20 components) the C3 and C4 electrodes independently achieved mean success rates of 94.63% and 94.73% for general movement vs. no movement, respectively, as shown in Figure 5 and Table A1. When the C3 and C4 electrodes were combined with the P3 electrode, as shown by the points for two and three electrodes in Figure 5, the success rates experienced varied outcomes, with decreases in some cases and stability and increases in others. However, for four electrodes, the success rates diminished, and this trend persisted for nine electrodes. This suggests that using a single electrode is preferable, as additional electrodes do not yield improvements. Furthermore, fewer electrodes reduce the time–computational costs of the algorithms and allow for higher algorithmic complexity. Based on these considerations, the decision was made to exclude four and nine electrodes from this work, opting for a single electrode, either C3 or C4, depending on the user, and considering the different motor imagery zones to test the performances of two and three electrodes.

The different motor imagery zones discussed in the Motor Imagery Brain Zone (MIZ) Section of Materials and Methods were tested using the P3, P4, T5, and T6 electrodes. Figure 7 shows that the P3 electrode had a better performance than the T5 electrode for all the subjects when compared with the C3 electrode. However, when compared with the C4 electrode, not all of the subjects had better P3 performance than T5 performance. The P4 and T6 electrodes are contraries, and the T6 electrode had a better performance than the T4 electrode with the C4 electrode in all subjects, but not with the C3 electrode.

According to the data, it seems that subject three has a left prominence while subject one has a right prominence, and subject two has the same prominence for the two sides, which is why these two electrodes are the main ones implicated in the motor imagery movements. Continuing with the focus on the subjects, the same zones do not have the same implications in the movements for the different subjects. Subject one is predominant in F8, F7, and P3 electrodes; subject two is predominant in T6, F7, and P3 electrodes; and subject three is predominant in P3, T6, and P4 electrodes.

The differentiation of the zones was tested using the far-away zones (FAZs) for the Cz and Pz electrodes in Figure 6. The different prominences remain out of scope when the C3 and C4 electrodes are involved because they unify both sides. The success rates when using these zones have similar results to those of some MIZ combinations (Figure 7). In fact, some electrodes selected for the MIZ have smaller success rates than those of some groups of FAZs. In most cases, across all tested users, the combination of two electrodes outperformed the combination of three electrodes, resulting in the decision to discard the use of three electrodes. When comparing one and two electrodes, the C3 and C4 electrodes consistently exhibited the highest success rates for all three subjects. Therefore, for this work, the use of two electrodes in combination was dismissed.

A preliminary user study is essential to determine individual preferences, and if a user requires more than one electrode, then the optimal zones must be selected based on specific tests.

### 4.4. Electrode-vs.-FastICACorr-Component Trade-Off

The success rate increases when the number of algorithm components increases. The best performance is for one electrode and 20 components. Adding electrodes generally decreases the success rate; consequently, the highest supported complexity for the algorithm is justified.

The best balance in the trade-off between the electrodes and algorithm components is 20 components for the C3 or C4 electrode.

### 4.5. Movement Differentiation

Finger motor imagery detection is feasible with one, two, three, and four electrodes with the C3 or C4 electrode and 20 components, yielding optimal results.

For 20 trials and ten components, the C4 electrode outperformed the C3 electrode, while, for 50 trials, the C3 electrode surpassed the C4 electrode, on average. However, this difference between 20 and 50 trials in the C3 electrode was not high, and for the other electrode groups, 20 trials was best; thus, the use of 50 trials was discarded for this movement differentiation.

Given that the C3 electrode is typically associated with the right hand, additional training is needed for an accurate focus on specific movements. Moreover, the success rate decreases with the inclusion of more electrodes.

For 20 trials and 20 components, the success rate was between 92% and 94% for all electrode groups. The C4 electrode was slightly better in terms of the mean, supporting the decision that one electrode was enough to obtain the best performance for this study.

As previously mentioned, 20 components consistently outperformed 10 components for each electrode group, which supports the idea that the weight of the components is more significant than the number of electrodes for the different movements.

These results also suggest that a specific movement corresponds to a specific zone within the MIZ because specific fingers, such as the thumb and pinkie, had better results with the C3–C4-electrode combination. The involvement of more than two electrodes that were within the common MIZ but far from their specific zones resulted in decreased success rates for all fingers.

### 4.6. Subject Independence and Requirements

Many hypotheses from [12] were reaffirmed in these experiments.

First, different subjects have different minds. Although the tasks and conditions were the same, it is not assumable that their brains worked in the same manner, which is called brain plasticity.

Subjects have differences in their success rates, starting with the dominant hand, necessitating adjustments for optimal success rates. These changes are not of significant concern because the wearable EEG will have a calibration phase to adapt to specific users.

Continuing with this hypothesis, distinct EEG signals arise from different subjects, emphasizing the importance of considering brain plasticity in the development of future devices.

A configurable wearable device that accommodates different subjects is crucial, ensuring scalability and mobility around the brain for diverse needs and reinforcing the idea of a comfortable and portable device for users.

## 5. Conclusions

This study comprehensively analyzed two algorithms, emphasizing the importance of making optimal design decisions for wearable EEG, considering the trade-off between performance and resource efficiency. The design decisions were obtained from the grouping characteristics discussed.

Regarding the sampling frequency, a system capable of obtaining data between 1 and 6.25 ms suffices for good success rates. Working within this margin depends on the device requirements imposed, such as the computational cost and battery life.

For general movement, 20 trials are adequate for good performance rates, while specific movements benefit from 50 trials over 20.

The FastICACorr algorithm was selected for continued testing on online cases, with 20 components selected due to the computational costs. This factor is inversely proportional to the number of electrodes: more components require fewer electrodes, and the weight of the components is greater than the weight of the electrodes in wearable EEG.

Depending on the requirements of the device and the user’s brain, the system will implement one electrode, either the C3 or C4 electrode. If a specific subject requires more electrodes, the C3 and C4 electrodes can be used together, and if additional electrodes are needed, the P3, P4, or T6 electrodes can be added.

A wearable device with these design decisions can be implemented to improve comfort and portability for users at any place and at any moment.

The general conclusion is that a few-electrode wearable EEG is feasible with algorithms that generate outputs in real execution time. Consequently, a portable and comfortable device for patients with specific requirements can be developed.

## Figures and Tables

**Table 1:** Success rates for one electrode for different databases (Fast Independent Component Analysis CORR algorithm).

DDBB (fs)	10 Components	20 Components
OODS (9.5 kHz)	84.80%	93.60%
NDS (1 kHz)	90.60%	95.10%
PDS (160 Hz)	92.00%	95.63%

**Table 2:** Success rates for one (C3 and C4), two (C3 and C4 together), and three (C3, C4, and P3) electrodes for 20 and 150 trials and three subjects for FB-CSSP algorithm.

Electrodes	C3		C4		C3, C4		C3, C4, P3	
Trials	20	150	20	150	20	150	20	150
Subject 1 (%)	100	100	100	100	100	100	100	100
Subject 2 (%)	100	100	100	100	100	100	100	100
Subject 3 (%)	100	100	100	100	100	100	100	100

**Table 3:** Success rates for two and four electrodes for far-away electrodes for FB-CSSP algorithm.

Electrodes	C3, Cz, C4, F7	C4, F7
Trials	20	20
Subject 1 (%)	100	100
Subject 2(%)	99.80	100
Subject 3 (%)	100	100

**Table 4:** Success rates for C3 electrode for different movements for 20 and 150 trials for FB-CSSPs algorithm.

Movement vs. No Movement	Thumb	Index	Middle	Ring	Pinkie
**Trials**	**20**				
**Subject 1 (%)**	60.7	52.4	65.5	60.7	72.6
**Subject 2 (%)**	61.9	67.9	61.9	59.5	56
**Subject 3 (%)**	59.5	58.3	57.1	73.8	52.4
**Trials**	**150**				
**Subject 1 (%)**	73.8	57.1	61.9	50	56
**Subject 2 (%)**	71.4	59.5	66.7	65.5	66.7
**Subject 3 (%)**	63.1	60.7	69	66.7	77.4

**Table 5:** Success rates for C4 electrode for different movements for 20 and 150 trials for FB-CSSPs algorithm.

Movement vs. No Movement	Thumb	Index	Middle	Ring	Pinkie
**Trials**	**20**				
**Subject 1 (%)**	70.2	64.3	71.4	50	63.1
**Subject 2 (%)**	57.1	61.9	50	50	56
**Subject 3 (%)**	53.6	64.3	61.9	54.8	58.3
**Trials**	**150**				
**Subject 1 (%)**	64.3	51.2	54.8	50	53.60
**Subject 2 (%)**	75	71.4	63.1	65.5	56
**Subject 3 (%)**	58.3	58.3	69	75	77.4

## Data Availability

Carretero Pérez, Ana (2024), “EEG Motor imagery open/close hands with C4 electrode dataset”, Mendeley Data, V1, https://doi.org/10.17632/bfn2pksz45.1 (accessed on 22 July 2024) [21].

## Appendix A

**Table A1:** Success rates for one electrode for different trials and components for FastICACorr algorithm.

Electrodes	C3					
Components	10				20	
Trials	20	50	100	150	20	50
Subject 1 (%)	86.8	90.8	88.6	88.8	95.5	93.9
Subject 2 (%)	90.5	87.3	87.5	87.5	94.7	94.8
Subject 3 (%)	88.8	87.7	87.2	87	93.7	93.9
Electrodes	C4					
Components	10				20	
Trials	20	50	100	150	20	50
Subject 1 (%)	87.8	89	89.3	89.1	93.1	94.9
Subject 2 (%)	90.6	88.1	88	88.1	95.1	95.1
Subject 3 (%)	88	89	87.8	87.6	94.5	94.8

**Table A2:** Success rates for two, three, and four electrodes for different trials and components for FastICACorr algorithm.

Electrodes	C3, C4			
Components	10		20	
Trials	20	50	20	50
Subject 1 (%)	87.7%	88.7%	93.6%	93.4%
Subject 2 (%)	88%	87.3%	94.6%	94.2%
Subject 3 (%)	87.3%	88.2%	91.7%	93.5%
Electrodes	C3, C4, P3			
Components	10		20	
Trials	20	50	20	50
Subject 1 (%)	88.8	88	93.2	93
Subject 2 (%)	89.3	86.3	94.3	94
Subject 3 (%)	87.5	87.8	92.9	92.8
Electrodes	C3, C4, P3, P4			
Components	10		20	
Trials	20	50	20	50
Subject 1 (%)	87.2	87.6	92.9	92.8
Subject 2 (%)	89.4	86.1	93.1	93.5
Subject 3 (%)	86.8	86.9	92	92.1

**Table A3:** Success rates for two electrodes for different trials and components for FastICACorr algorithm.

Electrodes	C3, C4, P3, P4, F3, F4, Cz, Pz, Fz
Components	20
Trials	20
Subject 1 (%)	92.7
Subject 2 (%)	92.6
Subject 3 (%)	90.9
